# Supplementary material for: An Engineered λ Phage Enables Enhanced and Strain-Specific Killing of Enterohemorrhagic Escherichia coli
Source: Microbiol Spectr. 2022 Jul 25;10(4):e01271-22. doi: 10.1128/spectrum.01271-22 (PMC9431524; doi:10.1128/spectrum.01271-22)
Supplement: Supplemental file 1 — Supplemental material. Download spectrum.01271-22-s0001.pdf, PDF file, 4.6 MB [file spectrum.01271-22-s0001.pdf]

Supplementary Figures

Fig. S1. Prevalence of eae gene and targeting site in EHEC strains

"O157:H7" indicates the EHEC strains with serotype O157:H7, and "other" indicates the EHEC strains with all serotypes (O26: H11, O121: H19, O45: H2 and other serotypes) except serotype O157:H7. “*eae* +” indicates strains that contain the *eae* gene, while “*eae* -” indicates strains that do not contain the *eae* gene. “Target site+” indicates strains that contain the *eae* gene, and can be targeted by the CRISPR-spacer, while “Target site-” indicates strains that either do not contain the *eae* gene, or the sequences of their *eae* genes do not match to the CRISPR-spacer.

Fig. S2 Construction and validation of the engineered lambda Phage (Eλ)

(**a**) Schematic diagram of phage engineering through fusion PCR; the primers are listed in Table S2 see Methods for details.

(**b**) Verification the engineered lambda phage Eλ using PCR. The locations and expected sizes of the PCR products are shown in (**a**); the PCR products of PCR1, PCR2, PCR3 and PCR4 confirm the genes of CRISPR-Cas3 system and crRNA were successfully incorporated into the Eλ Phage.

(**c**) PCR results of the *cro* gene in the wildtype (left) and engineered phage (right).

(**d**) PCR products covering the whole engineered region in the wildtype (right) and engineered (left) phages. The corresponding primer sequences can be found in Table S2.

Fig. S3. Plaque formation analysis of EHEC and EPEC strains infected by wtλ and Eλ phages

The EHEC strains (or EPEC strains) were incubated with 10-fold serial dilutions (10^5, 10^6, 10^7, 10^8, and 10^9 PFU) of wtλ (or Eλ) particles at a ratio of 1:1 on double-layer LB agar (0.8% agar on the top layer) for 6 hours at 37 °C.

Fig. S4. In vitro antibacterial experiments of wtλ and Eλ phages

(**a**) The antibacterial curves of wtλ at four different MOIs.

(**b**) The antibacterial curves of Eλ at four different MOIs.

(**c**) Antibacterial curve of Eλ against recovered EHEC at MOI=10 *in vitro* (n=3).

(**d**) Killing test of the wtλ against 22 *Escherichia coli* strains, including three EHEC strains, four EPEC strains, five laboratory strains and ten gut commensal strains isolated from human feces. “Target site+” indicates strains that contain the *eae* gene, and can be targeted by the CRISPR-spacer, while “Target site-” indicates strains that either do not contain the *eae* gene, or the sequences of their *eae* genes do not match to the CRISPR-spacer. The survival rate was measured at the 12th

hour after infection with a MOI (multiplicity of infection) of 10, and calculated as CFU (with wtλ)/CFU (without wtλ). (n = 3). All of the data are expressed as the mean±SD.

Fig. S5. Eλ phage alleviated EHEC-induced tissues damage

(**a**) Colon length measured from randomly selected mice from the four experimental groups, including the EHEC, EHEC+wtλ, and EHEC+Eλ and Control (without infection) groups; one random representative was chosen from each group. All mice were sacrificed on day 4; colons were isolated on the same day.

(**b**) Representative images of hematoxylin and eosin (H&E) stained tissues including kidney and liver; the tissues were taken from mice of day 4. Magnification: 200×; Bars: 100 μm. In HE-stained sections of kidney, red arrows indicate tubular epithelial cell necrosis; black arrows indicate mild watery degeneration of tubular epithelial cells; blue arrows indicate medullary vascular congestion in kidney medulla. In HE-stained sections of the liver, black arrows indicate hepatocyte necrosis and lymphocyte infiltration blue arrows indicate mild degeneration and edema of the hepatocytes.

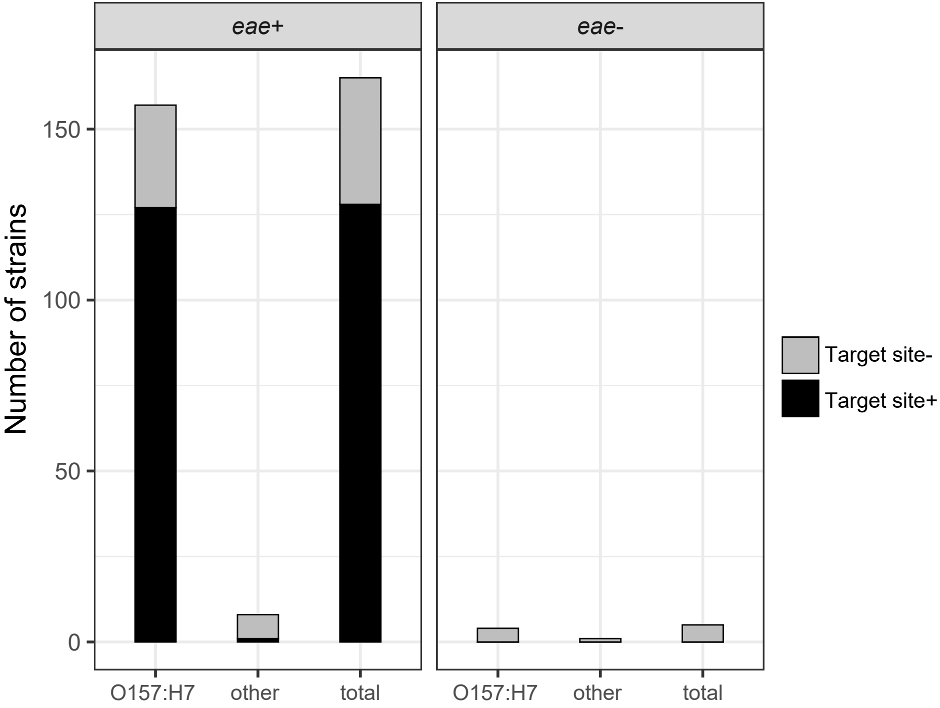

eae+
eae-
Number of strains
150
100
50
0
O157:H7
other
total
O157:H7
other
total
Target site-
Target site+

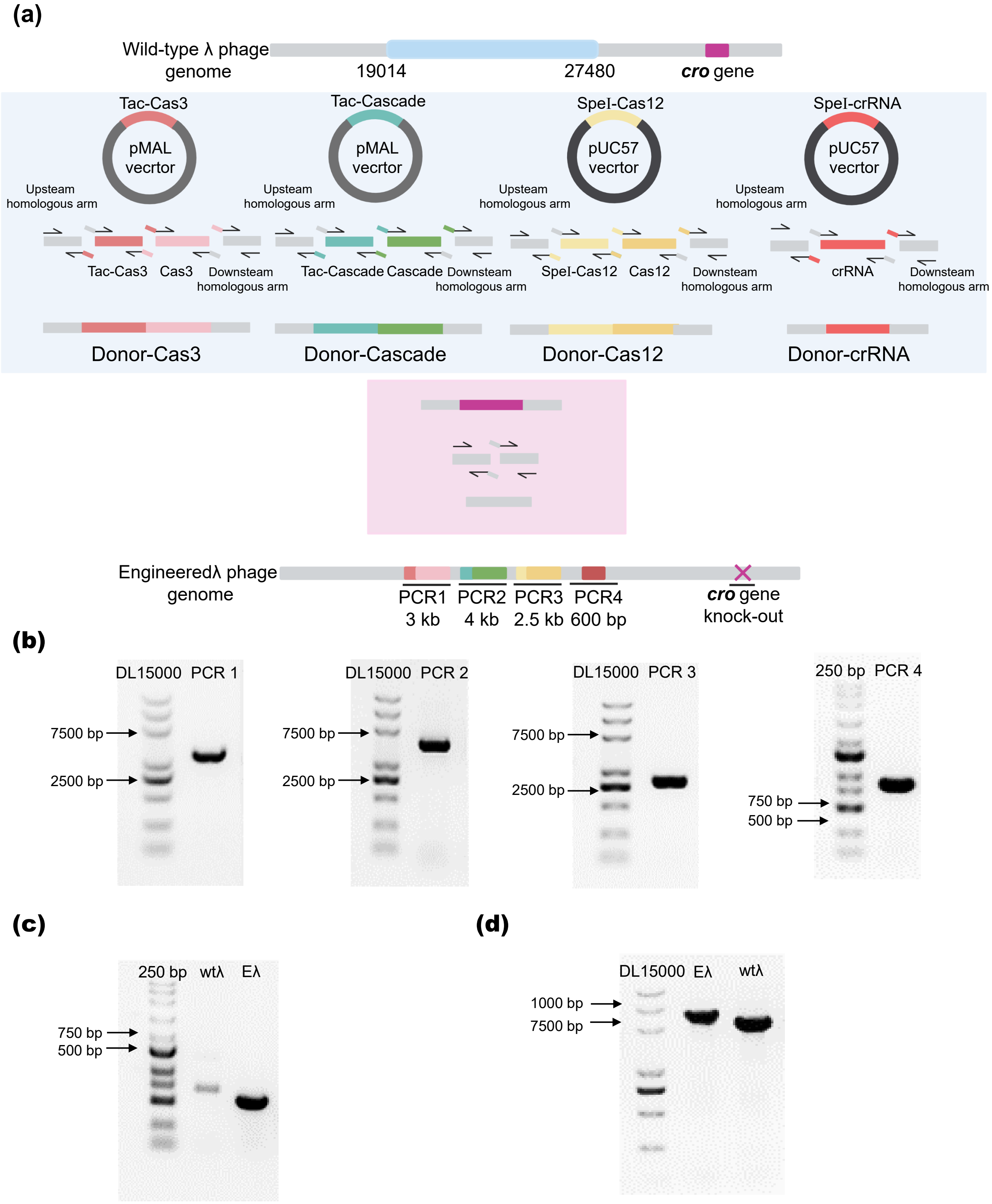

(a)
Wild-type λ phage genome
19014
27480
cro gene
Tac-Cas3
pMAL vecrtor
Tac-Cascade
pMAL vecrtor
SpeI-Cas12
pUC57 vecrtor
SpeI-crRNA
pUC57 vecrtor
Upsteam homologous arm
Tac-Cas3
Cas3
Downsteam homologous arm
Tac-Cascade
Cascade
SpeI-Cas12
Cas12
crRNA
Donor-Cas3
Donor-Cascade
Donor-Cas12
Donor-crRNA
Engineeredλ phage genome
PCR1 3 kb
PCR2 4 kb
PCR3 2.5 kb
PCR4 600 bp
cro gene knock-out
(b)
DL15000
PCR 1
PCR 2
PCR 3
250 bp
PCR 4
7500 bp
2500 bp
750 bp
500 bp
(c)
250 bp
wtλ
Eλ
750 bp
500 bp
(d)
DL15000
Eλ
wtλ
1000 bp
7500 bp

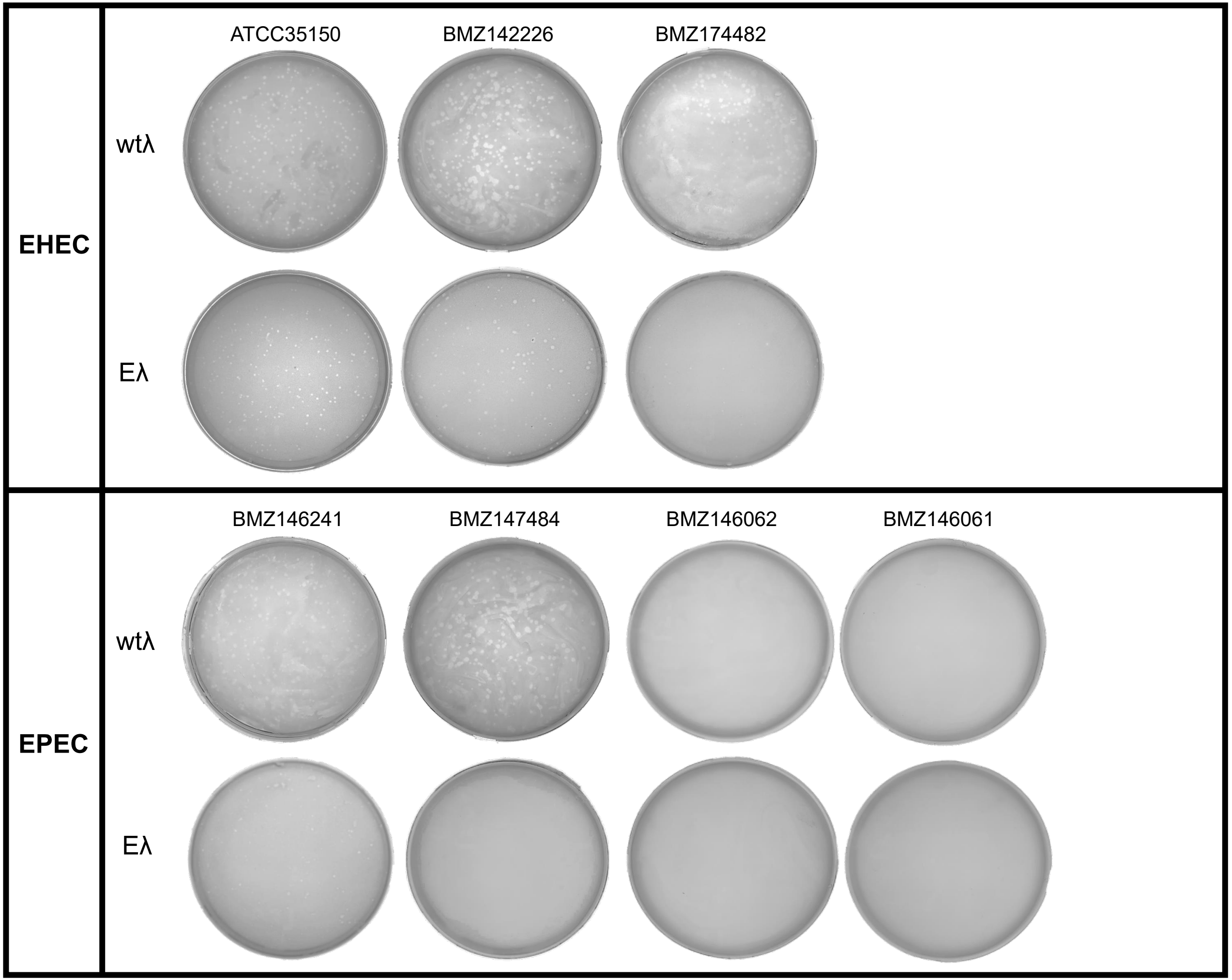
EHEC
ATCC35150
BMZ142226
BMZ174482
wtλ
Eλ
EPEC
BMZ146241
BMZ147484
BMZ146062
BMZ146061
wtλ
Eλ

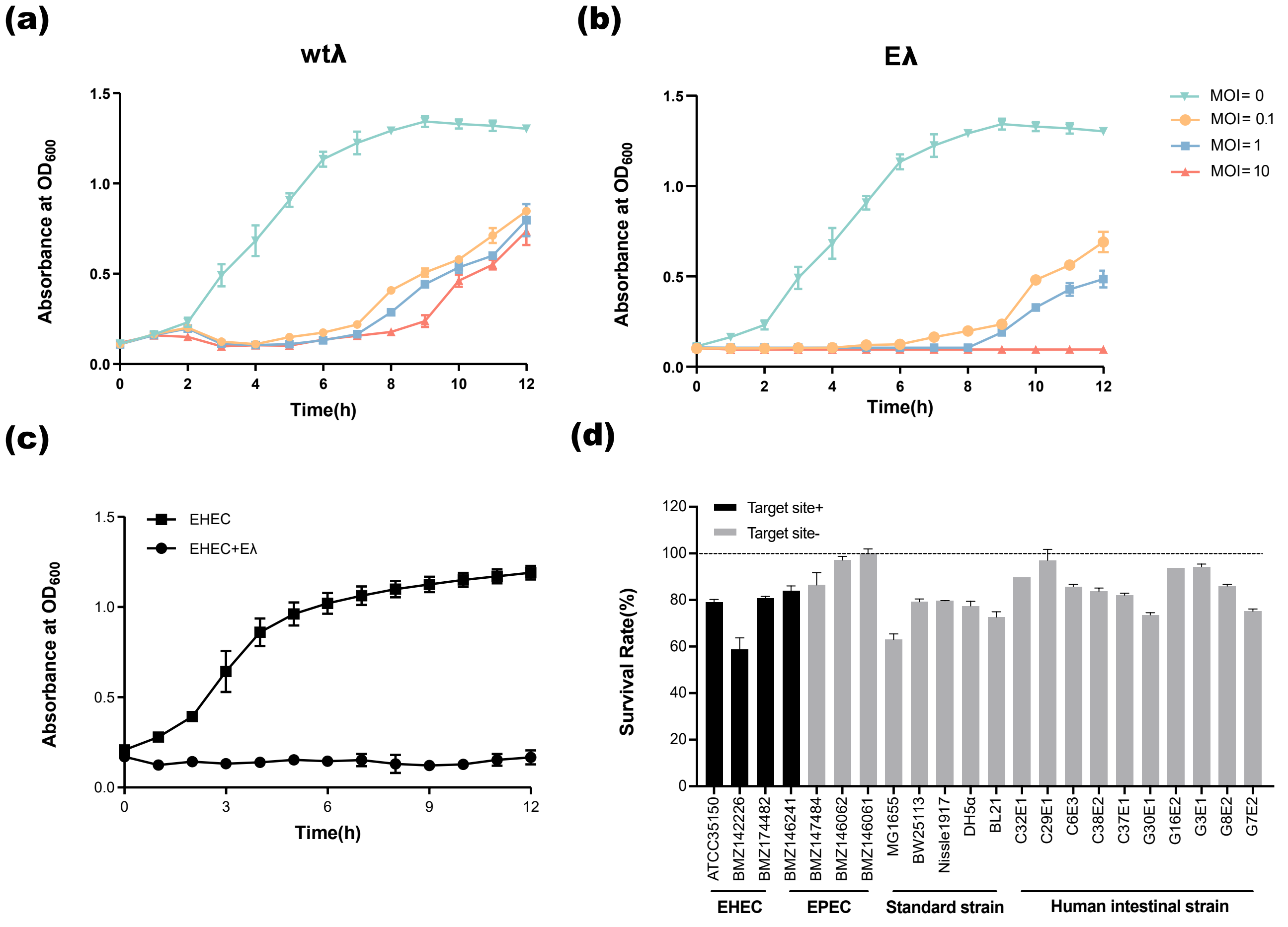
(a)
wtλ
Absorbance at OD600
Time(h)
(b)
Eλ
MOI= 0
MOI= 0.1
MOI= 1
MOI= 10
Absorbance at OD600
Time(h)
(c)
EHEC
EHEC+Eλ
Absorbance at OD600
Time(h)
(d)
Target site+
Target site-
Survival Rate(%)
ATCC35150
BMZ142226
BMZ174482
BMZ146241
BMZ147484
BMZ146062
BMZ146061
MG1655
BW25113
Nissle1917
DH5α
BL21
C32E1
C29E1
C6E3
C38E2
C37E1
G30E1
G16E2
G3E1
G8E2
G7E2
EHEC
EPEC
Standard strain
Human intestinal strain

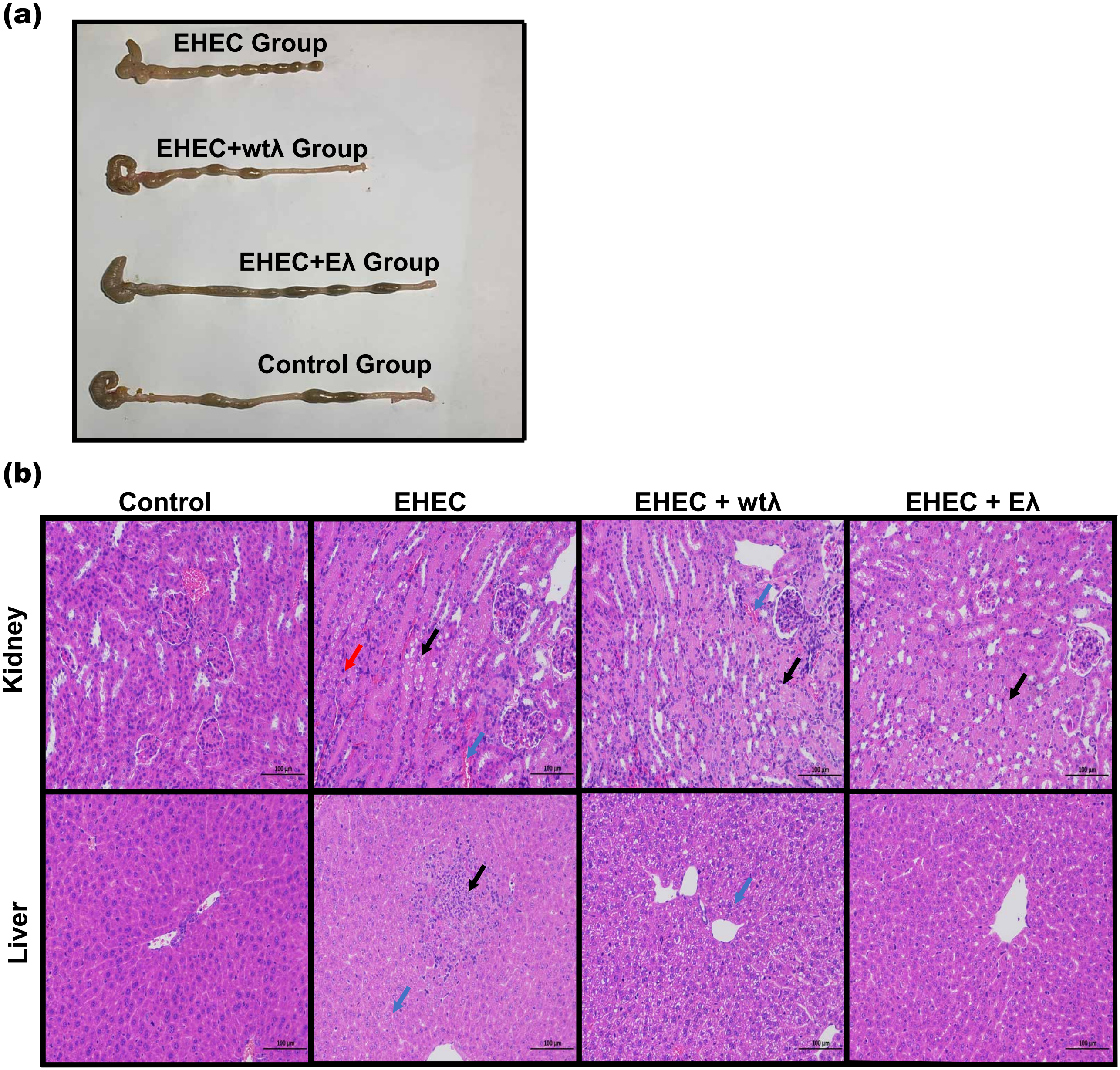

(a)
EHEC Group
EHEC+wtλ Group
EHEC+Eλ Group
Control Group
(b)
Control
EHEC
EHEC + wtλ
EHEC + Eλ
Kidney
Liver

Supplementary Tables

Table S1. Prevalence of eae gene and targeting site in EHEC strains

GenBank	eae	target site	serotypes
GCA_000006665.1	√	×	O157:H7
GCA_000008865.2	√	×	O157:H7
GCA_000010745.1	√	×	O103:H2
GCA_000021125.1	√	×	O157:H7
GCA_000022225.1	√	×	O157:H7
GCA_000091005.1	√	×	O26:H11
GCA_000730345.1	√	×	O157:H7
GCA_000732965.1	√	×	O157:H7
GCA_000803705.1	√	×	O157:H7
GCA_001307215.1	√	×	O157:H7
GCA_001651925.2	√	×	O157:H7
GCA_001651945.2	√	×	O157:H7
GCA_001651965.2	√	×	O157:H7
GCA_001695515.1	√	×	O157:H7
GCA_002844165.1	√	×	O121:H19
GCA_003722195.1	√	×	O157:H7
GCA_003966795.1	√	×	O157:H7
GCA_004118915.1	√	×	O157:H7
GCA_005037715.2	√	×	O121:H19
GCA_005037725.2	√	×	O26:H11
GCA_005037795.2	√	×	O103:H2
GCA_005037845.2	√	×	O45:H2
GCA_006514375.1	√	×	O157:H7
GCA_007922655.1	√	×	O157:H7
GCA_009627495.1	√	×	O157:H7
GCA_009650175.1	√	×	O157:H7
GCA_009931235.1	√	×	O157:H7
GCA_013167795.1	√	×	O157:H7
GCA_013167815.1	√	×	O157:H7
GCA_013168255.1	√	×	O157:H7
GCA_016834635.1	√	×	O157:H7

GCA_016834675.1	√	×	O157:H7
GCA_016834695.1	√	×	O157:H7
GCA_016834735.1	√	×	O157:H7
GCA_016835055.1	√	×	O157:H7
GCA_017357545.1	√	×	O157:H7
GCA_022869945.1	√	×	O157:H7
GCA_000155125.1	√	√	O157:H7
GCA_000978815.2	√	√	O157:H7
GCA_000978845.2	√	√	O157:H7
GCA_001558995.2	√	√	O157:H7
GCA_001753445.1	√	√	O157:H7
GCA_001753465.1	√	√	O157:H7
GCA_001753485.1	√	√	O157:H7
GCA_001753505.1	√	√	O157:H7
GCA_001753525.1	√	√	O157:H7
GCA_001753545.1	√	√	O157:H7
GCA_001753565.1	√	√	O157:H7
GCA_001865295.1	√	√	O157:H7
GCA_005037735.2	√	√	O157:H7
GCA_005885915.1	√	√	O157:H7
GCA_005885955.1	√	√	O157:H7
GCA_013167135.1	√	√	O157:H7
GCA_013167155.1	√	√	O157:H7
GCA_013167175.1	√	√	O157:H7
GCA_013167195.1	√	√	O157:H7
GCA_013167235.1	√	√	O157:H7
GCA_013167275.1	√	√	O157:H7
GCA_013167295.1	√	√	O157:H7
GCA_013167315.1	√	√	O157:H7
GCA_013167335.1	√	√	O157:H7
GCA_013167355.1	√	√	O157:H7
GCA_013167375.1	√	√	O157:H7
GCA_013167395.1	√	√	O157:H7
GCA_013167415.1	√	√	O157:H7
GCA_013167435.1	√	√	O157:H7
GCA_013167455.1	√	√	O157:H7
GCA_013167475.1	√	√	O157:H7
GCA_013167495.1	√	√	O157:H7
GCA_013167515.1	√	√	O157:H7

GCA_013167535.1	√	√	O157:H7
GCA_013167555.1	√	√	O157:H7
GCA_013167575.1	√	√	O157:H7
GCA_013167595.1	√	√	O157:H7
GCA_013167615.1	√	√	O157:H7
GCA_013167635.1	√	√	O157:H7
GCA_013167655.1	√	√	O157:H7
GCA_013167675.1	√	√	O157:H7
GCA_013167695.1	√	√	O157:H7
GCA_013167715.1	√	√	O157:H7
GCA_013167735.1	√	√	O157:H7
GCA_013167755.1	√	√	O157:H7
GCA_013167775.1	√	√	O157:H7
GCA_013167875.1	√	√	O157:H7
GCA_013168035.1	√	√	O157:H7
GCA_013168055.1	√	√	O157:H7
GCA_013168075.1	√	√	O157:H7
GCA_013168095.1	√	√	O157:H7
GCA_013168115.1	√	√	O157:H7
GCA_013168135.1	√	√	O157:H7
GCA_013168155.1	√	√	O157:H7
GCA_013168175.1	√	√	O157:H7
GCA_013168195.1	√	√	O157:H7
GCA_013168215.1	√	√	O157:H7
GCA_013168235.1	√	√	O157:H7
GCA_013343595.1	√	√	O157:H7
GCA_013343635.1	√	√	O157:H7
GCA_015353135.1	√	√	O157:H7
GCA_016458885.1	√	√	O157:H7
GCA_016458905.1	√	√	O157:H7
GCA_016458925.1	√	√	O157:H7
GCA_016458945.1	√	√	O157:H7
GCA_016834715.1	√	√	O157:H7
GCA_016834755.1	√	√	O157:H7
GCA_016834775.1	√	√	O157:H7
GCA_016834795.1	√	√	O157:H7
GCA_016834815.1	√	√	O157:H7
GCA_016834835.1	√	√	O157:H7
GCA_016834855.1	√	√	O157:H7

GCA_016834875.1	√	√	O157:H7
GCA_016834895.1	√	√	O157:H7
GCA_016834915.1	√	√	O157:H7
GCA_016834935.1	√	√	O157:H7
GCA_016834955.1	√	√	O157:H7
GCA_016834975.1	√	√	O157:H7
GCA_016834995.1	√	√	O157:H7
GCA_016835015.1	√	√	O157:H7
GCA_016835035.1	√	√	O157:H7
GCA_016835075.1	√	√	O157:H7
GCA_016835095.1	√	√	O157:H7
GCA_017164755.1	√	√	O157:H7
GCA_017164775.1	√	√	O157:H7
GCA_017164795.1	√	√	O157:H7
GCA_017164815.1	√	√	O157:H7
GCA_017164835.1	√	√	O157:H7
GCA_017164855.1	√	√	O157:H7
GCA_017164875.1	√	√	O157:H7
GCA_017164895.1	√	√	O157:H7
GCA_017164915.1	√	√	O157:H7
GCA_017164935.1	√	√	O157:H7
GCA_017164955.1	√	√	O157:H7
GCA_017164975.1	√	√	O157:H7
GCA_017164995.1	√	√	O157:H7
GCA_017165015.1	√	√	O157:H7
GCA_017165035.1	√	√	O157:H7
GCA_017165055.1	√	√	O157:H7
GCA_017165075.1	√	√	O157:H7
GCA_017165095.1	√	√	O157:H7
GCA_017165115.1	√	√	O157:H7
GCA_017165135.1	√	√	O157:H7
GCA_017165155.1	√	√	O157:H7
GCA_017165215.1	√	√	O157:H7
GCA_017165235.1	√	√	O157:H7
GCA_017165255.1	√	√	O157:H7
GCA_017165275.1	√	√	O157:H7
GCA_017165295.1	√	√	O157:H7
GCA_017165315.1	√	√	O157:H7
GCA_017165335.1	√	√	O157:H7

GCA_017165355.1	√	√	O157:H7
GCA_017165375.1	√	√	O157:H7
GCA_017165395.1	√	√	O157:H7
GCA_017165415.1	√	√	O157:H7
GCA_017165435.1	√	√	O157:H7
GCA_017165455.1	√	√	O157:H7
GCA_020423145.1	√	√	O157:H7
GCA_020423165.1	√	√	O157:H7
GCA_020423185.1	√	√	O157:H7
GCA_020423205.1	√	√	O157:H7
GCA_020423225.1	√	√	O157:H7
GCA_021166395.1	√	√	O157:H7
GCA_021166415.1	√	√	O157:H7
GCA_021166435.1	√	√	O157:H7
GCA_021184105.1	√	√	O157:H7
GCA_021184125.1	√	√	O157:H7
GCA_003028715.1	×	×	O157:H7
GCA_003028755.1	×	×	O157:H7
GCA_005037775.2	×	×	O91:H21
GCA_011068385.1	×	×	O157:H7
GCA_022558925.1	×	×	O157:H7
GCA_005037815.2	√	√	O145: NM

"✓" indicates that the strain contains the corresponding gene or sequence, and "×" indicates that it does not.

Table S2. Bacterial strains and plasmids used in this study

Strain or plasmid	Source or reference	*eae* gene	Target site
E. coli EHEC ATCC35150	Mingzhoubio (Ningbo, China, https://www.mingzhoubio.com/)	✓	✓
E. coli EHEC BMZ142226	Mingzhoubio (Ningbo, China, https://www.mingzhoubio.com/)	✓	✓
E. coli EHEC BMZ174482	Mingzhoubio (Ningbo, China, https://www.mingzhoubio.com/)	✓	✓
E. coli EPEC BMZ146241	Mingzhoubio (Ningbo, China, https://www.mingzhoubio.com/)	✓	✓
E. coli EPEC BMZ147484	Mingzhoubio (Ningbo, China, https://www.mingzhoubio.com/)	✓	×
E. coli EPEC BMZ146062	Mingzhoubio (Ningbo, China, https://www.mingzhoubio.com/)	✓	×
E. coli EPEC BMZ146061	Mingzhoubio (Ningbo, China, https://www.mingzhoubio.com/)	✓	×
E. coli CCTCC AB2013329	China Center For Typr Culture Collection (http://cctcc.whu.edu.cn/)	×	×
AB2013329-λΔcro	This study	×	×
AB2013329-λΔcro::Cas3	This study	×	×
AB2013329-λΔcro::Cas3::Cascade	This study	×	×
AB2013329-λΔcro::Cas3::Cascade::crRNA	This study	×	×
E. coli MG1655	(1)	×	×
E. coli DH5α	(2)	×	×
E. coli BW25113	(2)	×	×
E. coli BL21	(2)	×	×
C32E1	This study	×	×
C29E1	This study	×	×
C6E3	This study	×	×
C38E2	This study	×	×
C37E1	This study	×	×
G30E1	This study	×	×
G16E2	This study	×	×
G3E1	This study	×	×
G8E2	This study	×	×
G7E2	This study	×	×

pTargetF	Miaolingbio (Wuhan, China, https://miaolingbio.biomart.cn/)
pCas	Miaolingbio (Wuhan, China, https://miaolingbio.biomart.cn/)
pUC57	Genscript (Nanjing, China, https://www.genscript.com.cn/)
pUC57-12	Genscript (Nanjing, China, https://www.genscript.com.cn/)
pMAL-c5x-TacCas3	Genscript (Nanjing, China, https://www.genscript.com.cn/)
pMAL-c5x-TacCascade	Genscript (Nanjing, China, https://www.genscript.com.cn/)

"✓" indicates that the strain contains the corresponding gene or sequence, and "×" indicates that it does not.

1. Hayashi K, Morooka N, Yamamoto Y, Fujita K, Isono K, Choi S, Ohtsubo E, Baba T, Wanner BL, Mori H, Horiuchi T. 2006. Highly accurate genome sequences of Escherichia coli K-12 strains MG1655 and W3110. Mol Syst Biol 2:2006.0007.

2. Tate H, Li C, Nyirabahizi E, Tyson GH, Zhao S, Rice-Trujillo C, Jones SB, Ayers S, M'Ikanatha N M, Hanna S, Ruesch L, Cavanaugh ME, Laksanalamai P, Mingle L, Matzinger SR, McDermott PF. 2021. A National Antimicrobial Resistance Monitoring System Survey of Antimicrobial-Resistant Foodborne Bacteria Isolated from Retail Veal in the United States. J Food Prot 84:1749-1759.

Table S3. Oligonucleotides used in this study

Oligonucleotide	Sequence (5′→3′)	Notes
sgCas3_1F	GAAGTACCGTTATGAGCTGAGTTTTAGAGCTAGAAATAGC	Amplify pTargetF plasmids containing sgRNA
sgCas3_1R	TCAGCTCATAACGGTACTTCACTAGTATTATACCTAGGAC	
sgCas3_2F	CAAATCGACGAATAACACGGGTTTTAGAGCTAGAAATAGC	
sgCas3_2R	CCGTGTTATTCGTCGATTTGACTAGTATTATACCTAGGAC	
sgCascade_1F	AAAACTGTTCCGGATCCGGGGTTTTAGAGCTAGAAATAGC	
sgCascade_1R	CCCGGATCCGGAACAGTTTT ACTAGTATTATACCTAGGAC	
sgCasAE_2F	ATGCTGAAATGAATTCTAAGGTTTTAGAGCTAGAAATAGC	
sgCasAE_2R	CTTAGAATTCATTTCAGCAT ACTAGTATTATACCTAGGAC	
sgCas12_1F	CATTCCAACCATCTGCTCGTGTTTTAGAGCTAGAAATAGC	
sgCas121R	ACGAGCAGATGGTTGGAATG ACTAGTATTATACCTAGGAC	
sgCas12F	GTTATGCAGGTCGTAGTGGGGTTTTAGAGCTAGAAATAGC	
sgCas12R	CCCACTACGACCTGCATAAC ACTAGTATTATACCTAGGAC	
sgcroF	AATGGTTGCATGTACTAAGGGTTTTAGAGCTAGAAATAGC	
sgcroR	CCTTAGTACATGCAACCATT ACTAGTATTATACCTAGGAC	
sgcrRNAF	ACCGACCGTCGGATAAGACGGTTTTAGAGCTAGAAATAGC	
sgcrRNAR	CGTCTTATCCGACGGTCGGTACTAGTATTATACCTAGGAC	
Hom_cro_UP_F	TGCTGCGGTAAGTCGCATAAAAAC	Donor- cro gene
Hom_cro_UP_R	TGATGCCCTTTACCGCCAGAGGTAAAATAGTCAACAC	
Hom_cro_DW_F	CTCTGGCGGTAAAGGGCATCAAATTAAACCACACCT	
Hom_cro_DW_R	TTTTCTGGCTGGTCAGAGGATTCG	
Hom_cr_UP_F	CGCTTTACCTTCGGTGTACAG	Donor- crRNA
Hom_cr_UP_R	AGAGGAATTCCCCACGAACGTCAGCGTCT	
pcrRNA_F	ACGTTCGTGGGGAATTCCTCTAGAGTCGACCTG	
pcrRNA_R	GGGATCGGCGGGGATCGGTCCAAAAAAC	
Hom_cr_DW_F	CGATCCCCGGGATTTCAGCGTCGGCGC	
Hom_cr_DW_R	GGCAATCCGGAACGATACCG	
T7_Cas3_F	AGTCTCGCGCTAAGGCGTTC	Donor- Cas3
T7_Cas3_R	AGGCAATGATAAATTAACAG	
T7_mg1655_Cas3F	AAGCTTGACGAAAGGAAATG	
T7_mg1655_Cas3R	GGGATTTGCAGGGATGACTC	
Tac_Cas3F	GGGATTAACCAGTCTCGCGCTAAGG	
Tac_Cas3R	ACTGTGAGCATGTTATTTGGGATTTGCAGGGATGACT	
Hom_Cas3_UP_F	AGCCAGTTTCTGGTTGC	
Hom_Cas3_UP_R	CGCGAGACTGGTTAATCCCTTTCAGATGACTCAC	
Hom_Cas3_DW_F	ATCCCAAATAACATGCTCACAGTCTGAG	

Hom_Cas3_DW_R	CATACAACGTTTCTGCGG	
Tac_Cascade_F	GGATACGGAAGCATTGACAATTAATCATCGGCTCGT	
Tac_Cascade_R	GGCGATCTCTCACAGTGGAGCCAAAGATAG	
T7_mg1655_Cascade F	TCCCTGTACGCCCGCGAAAC	
T7_mg1655_Cascade R	CACAGTGGAGCCAAAGATAG	Donor- Cascade
Hom_Cascade_UP_F	AGGGGCCGCGGG	
Hom_Cascade_UP_R	GATTAATTGTCAATGCTTCCGTATCCTTCACC	
Hom_Cascade_DW_F	CCACTGTGAGAGATCGCCTAGTGATTTTAAACT	
Hom_Cascade_DW_R	TCCTTTGTTACAATGTTTTATCTTACTGT	
Spe1_Cas12_F	GAATAGTCATTCCGGAATTCCTCTAGAGTCGACC	
Spe1_Cas12_R	AAGCCAGGTCATTTTATTAGGTCAAACAGGTAAAAAAGAC	
Hom_Cas12_UP_F	TCCAATATAAAAGTATTGTGTACCTTTTG	Donor- Cas12
Hom_Cas12_UP_R	CTAGAGGAATTCCGGAATGACTATTCCTTTGTTACAATG	
Hom_Cas12_DW_F	GAGTAGCTGAGCACACAAAGCTTTGCAC	
Hom_Cas12_DW_R	ATCTTTATGTCGAGCAAAGCAA	
LMDYZF	CGGGCGTTTTATTATAAAACAGTGAGAGG	Amplify lambda phage
LMDYZR	AGCACAAAGCCTCGCAATC	
Cas9yz_F	CGACTCGGTGCCACTTTTTC	Amplify pCas plasmids
Cas9yz _R	CAGTGAGCGCAACGCAATTA	
crRNA expression Cassette	GGAATTCCTCTAGAGTCGACCTGCAGAAGCTTGAAGATCTTCATTACC<br>CTTTGACAGCTAGCTCAGTCCTAGGTATAATACTAGTGAGTTCCCCGC<br>GCCAGCGGGGATAAACCGCTAACGGTAAGGCAACCGTAACGTTGAA<br>GTCGGAGTTCCCCGCGCCAGCGGGGATAAACCGCTAACGGTAAGGC<br>AACCGTAACGTTGAAGTCGGAGTTCCCCGCGCCAGCGGGGATAAAC<br>CGCTAACGGTAAGGCAACCGTAACGTTGAAGTCGGAGTTCCCCGCG<br>CCAGCGGGGATAAACCGCTAACGGTAAGGCAACCGTAACGTTGAAGT<br>CGGAGTTCCCCGCGCCAGCGGGGATAAACCGCTAACGGTAAGGCAA<br>CCGTAACGTTGAAGTCGGAGTTCCCCGCGCCAGCGGGGATAAACCG<br>CTAACGGTAAGGCAACCGTAACGTTGAAGTCGGAGTTCCCCGCGCCA<br>GCGGGGATAAACCGCTAGCATAACCCCTTGGGGCCTCTAAACGGGT<br>CTTGAGGGGTTTTTTGGACCGATCCCCG	SpeI promoter, the repeats and spacer are highlighted green, yellow and red, respectively.

Table S4. Oligonucleotides and templates used for construction of bacteria, phages and plasmids

Constructed Phage/plasmid	Oligonucleotides for PCR	DNA template
pTarget F-sgRNA	sgRNA_F/R	pTarget F
TacCas3 DNA	T7_Cas3_F/R	pMAL-c5x-TacCas3
Cas3 DNA	T7_mg1655_Cas3F/R	Genomic DNA of MG1655
Tac-Cas3 DNA	Tac_Cas3F/R	TacCas3 DNA, Cas3 DNA
HU_Cas3 DNA	Hom_Cas3_UP_F/R	Genomic DNA of lambda phage
HD_Cas3 DNA	Hom_Cas3_DW_F/R	Genomic DNA of lambda phage
Donor-Cas3 DNA	Hom_Cas3_UP_F/Hom_Cas3_DW_R	Tac-Cas3 DNA, HU_Cas3 DNA, HD_Cas3 DNA
Tac-Cascade DNA	Tac_Cascade_F/R	pMAl-c5x-TacCascade, Genomic DNA of MG1655
HU_Cascade DNA	Hom_Cascade_UP_F/R	Genomic DNA of lambda phage
HD_Cascade DNA	Hom_CascadeE_DW_F/R	Genomic DNA of lambda phage
Donor-Cascade DNA	Hom_Cascade_UP_F/Hom_CasAE_DW_R	Tac-Cascade DNA, HU_Cascade DNA, HD_Cascade DNA
SpeI-Cas12 DNA	Spe1_Cas12_F	pUC57-12, Genomic DNA of MG1655
HU_Cas12 DNA	Hom_Cas12_UP_F/R	Genomic DNA of lambda phage
HD_Cas12 DNA	Hom_Cas12_DW_F/R	Genomic DNA of lambda phage
Donor-Cas12 DNA	Hom_Cas12_UP_F/Hom_Cas12_DW_R	SpeI-Cas12 DNA, HU_Cas12 DNA, HD_Cas12 DNA
crRNA DNA	pcrRNA_F/R	pUC57-eae
HU_cr DNA	Hom_cr_UP_F/R	Genomic DNA of lambda phage
HD_cr DNA	Hom_cr_DW_F/R	Genomic DNA of lambda phage
Donor crRNA DNA	Hom_cr_UP_F/R	crRNA DNA, HU_cr DNA, HD_cr DNA
HU_cro DNA	Hom_cro_UP_F/R	Genomic DNA of lambda phage
HD_cro DNA	Hom_cro_DW_F/R	Genomic DNA of lambda phage
Donor cro DNA	Hom_cro_UP_F/Hom_cro_DW_R	HU_cro DNA, HD_cro DNA

Table S5. Mouse Fecal 16s DNA Sequencing Information

Sample name	Experiment/Run title	Accession	Library Construction /Experimental Design	Group	Description
EWTP1	EG_EWTP1	SAMC704341	DNA for each sample was extracted with Power Fecal® DNA Isolation Kit DNA (Qiagen), then V3-V4 (338F-806R) of 16S rDNA was amplified. Finally, the amplicons were sequenced on Illumina NovaSeq 6000 platform in pair-end 250 bp mode.	EHEC + wtλ treatment group	Intraperitoneal injection of mitomycin and gavage of enterohemorrhagic Escherichia coli (EHEC); gavage of wild-type λ phage 24 hours later
EWTP2	EG_EWTP2	SAMC704342			
EWTP3	EG_EWTP3	SAMC704343			
EWTP4	EG_EWTP4	SAMC704344			
EH1	EG_EH1	SAMC704345		EHEC group	Intraperitoneal injection of mitomycin and gavage of EHEC
EH2	EG_EH2	SAMC704346			
EH3	EG_EH3	SAMC704347			
EH4	EG_EH4	SAMC704348			
CT1	EG_CT1	SAMC704349		Control group	Intraperitoneal injection of mitomycin
CT2	EG_CT2	SAMC704350			
CT3	EG_CT3	SAMC704351			
CT4	EG_CT4	SAMC704352			
EEP1	EG_EEP1	SAMC704353		EHEC + Eλ treatment group	Intraperitoneal injection of mitomycin and gavage of EHEC; gavage of engineered λ phage 24 hours later
EEP2	EG_EEP2	SAMC704354			
EEP3	EG_EEP3	SAMC704355			
EEP4	EG_EEP4	SAMC704356			
